# Supplementary material for: Movi Color: fast and accurate long-read classification with the move structure
Source: bioRxiv. 2025 May 27:2025.05.22.655637. Preprint. [Version 1] doi: 10.1101/2025.05.22.655637 (PMC12154825; doi:10.1101/2025.05.22.655637)
Supplement: Supplement 1 [file NIHPP2025.05.22.655637v1-supplement-1.pdf]

bioRxiv preprint doi: https://doi.org/10.1101/2025.05.22.655637; this version posted May 27, 2025. The copyright holder for this preprint (which was not certified by peer review) is the author/funder, who has granted bioRxiv a license to display the preprint in perpetuity. It is made available under aCC-BY 4.0 International license.

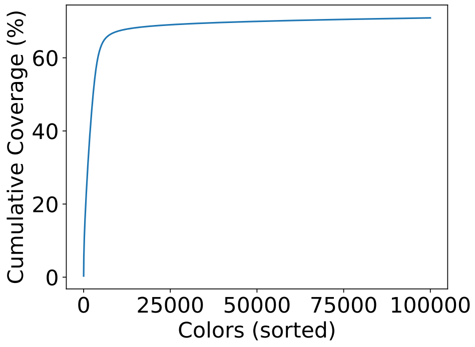

Supplementary Figure 1 The cumulative coverage (% of runs) of the 10,000 most frequent colors (out of over 2.4 billion total colors) in index of *Pseudomonadota* reference genomes. The first 10,000 most frequent colors cover 67.3% of the runs.

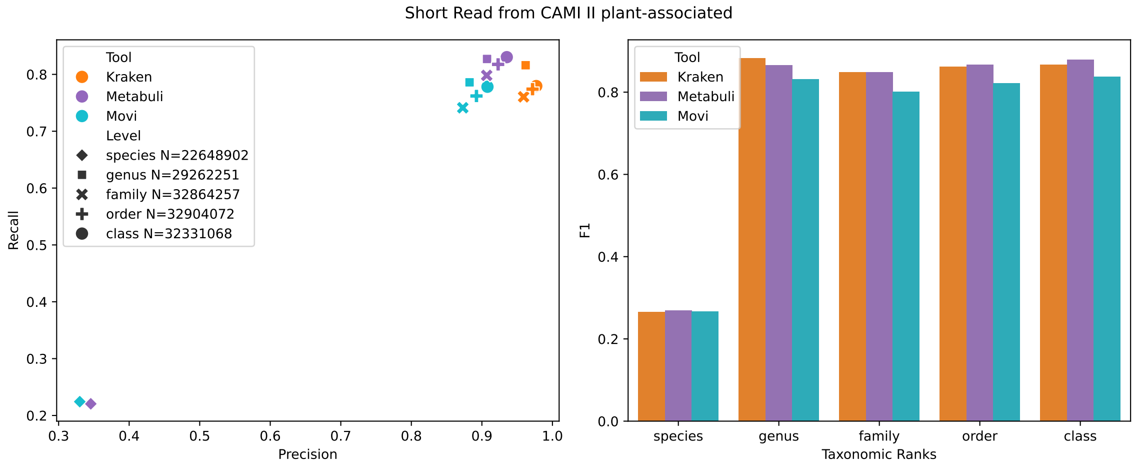

Supplementary Figure 2 Classification accuracies of Movi Color compared to Metabuli and Kraken 2 on short reads.
